# Unraveling the Impact of pH on the Crystallization of Pharmaceutical Proteins: A Case Study of Human Insulin

**DOI:** 10.1021/acs.cgd.1c01463

**Published:** 2022-04-12

**Authors:** Frederik J. Link, Jerry Y. Y. Heng

**Affiliations:** † Department of Chemical Engineering, Imperial College London, South Kensington Campus, London SW7 2AZ, U.K.; ‡ Institute for Molecular Science and Engineering, Imperial College London, South Kensington Campus, London SW7 2AZ, U.K.

## Abstract

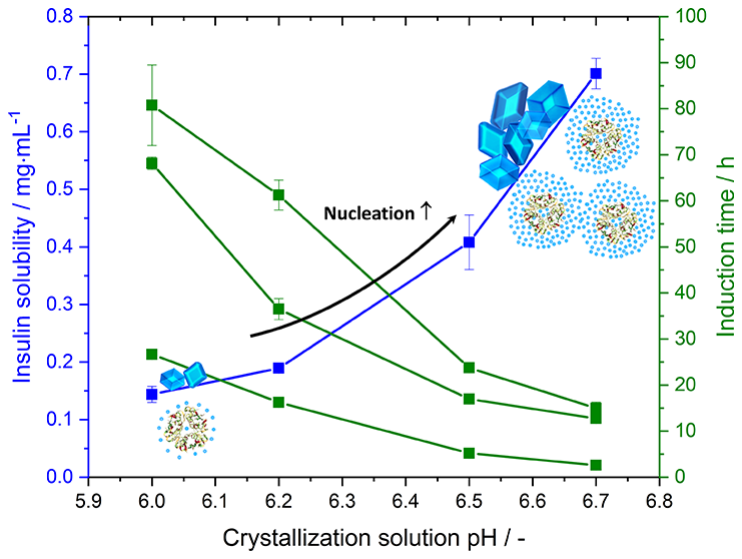

One of the most crucial parameters in protein crystallization is pH, as it governs the protein’s electrostatic interactions. However, the fundamental role of pH on crystallization still remains unknown. Here, we systematically investigated the crystallization of human insulin (isoelectric point 5.3) at various pHs between 6.0 and 6.7 at different supersaturation ratios, up to 20.9. Our results demonstrate that the pH has an opposing effect on solubility and nucleation rate as a shift in pH toward a more basic milieu increases the solubility by 5-fold while the onset of nucleation was accelerated by a maximum of 8.6-fold. To shed light on this opposing effect, we evaluated the protein–protein interactions as a function of pH by measuring the second virial coefficient and hydrodynamic radius and showed that a change in pH of less than one unit has no significant impact on the protein–protein interactions. As it is widely understood that the increase in protein solubility as a function of pH is due to the increase in the repulsive electrostatic interactions, we have demonstrated that the increase in insulin solubility and decrease in the onset of nucleation are independent of the protein–protein interactions. We hypothesize that it is the electrostatic interactions between both ions and solvent molecules and the protein residues that are governing the crystallization of human insulin. The findings of this study will be of crucial importance for the design of novel crystallization pathways.

## Abstract 2

We show experimentally that shifting the pH toward a more basic milieu increases the solubility but more importantly enhances the nucleation of the acidic protein human insulin. Studying the second virial coefficient and hydrodynamic radius revealed that the protein−protein interactions are independent of the pH and hence we hypothesize that it is the water structuring around the protein’s surface that plays a crucial role in crystallization.

## Introduction

Protein-based biological products have emerged as the next-generation of pharmaceuticals owing to their high selectivity toward their target and high potency with increased safety, efficacy, and tolerability in humans.^1,2^ Crystallization of these proteins is mainly carried out to obtain structural data, which is needed for protein engineering, understanding structure–activity relationships, or for pharmaceutical formulations. Due to the complexity of proteins and the limited understanding of the underlying physico–chemical interactions, such as hydrogen bonding, electrostatic, and hydrophobic interactions between the protein molecules, as well as between proteins and other solvent molecules or ions in solution, the designing of suitable crystallization pathways relies on intensive screening and trial-and-error approaches.^3,4^ Not only the magnitude but also the orientation of these interactions is crucial in successful protein crystallization.^5−7^ Nucleation is the critical and rate-limiting step of the crystallization process, which is affected by both thermodynamic and kinetic factors.^8,9^ While more than 30 different factors have been reported to impact nucleation,^4^ the most known factors are temperature,^10^ solution pH,^6,7,11^ type and concentration of salt^12,13^ or additives,^14^ and degree of supersaturation.^15^ A common crystallization pathway is salting-out, in which buffer concentration or salt type is changed^11,12,16^ or even additional salts are added.^13,17,18^ This results in the disruption of the water structure around the protein’s surface leading to a facilitated crystallization. Salting-out is not always a feasible approach to obtain protein crystals, hence, additives or organic solvents^11,19,20^ have been found to successfully enhance protein crystallization by stabilizing the protein and successfully disturbing the water layer in the vicinity of the protein’s surface. Understanding the underlying mechanism of protein crystallization has been the focus of research for years, and both computational and experimental approaches have been developed to shed light on how these factors impact crystallization.^13,17,19−23^

Being one of the most important crystallization factors, the pH of the crystallization cocktail prior to crystallization is quite often not measured, inaccurately determined, or not even reported. This lack of sufficient information on the pH of the crystallization cocktail stems from the small volume (∼μL) in which crystallization is normally executed for protein crystallography, which does not allow the measurement of the pH in the crystallization cocktail (e.g., after mixing the protein solution, precipitant solution, additional solvent, or additive solution). Quite often, the pH of the final crystallization cocktail is different from the pH of the stock protein solution because the addition of additives, precipitants, or additional solvents impacts the pH.^11,20^

However, the overall determination of the pH of the crystallization cocktail is crucial, as it modulates the net surface charge on the protein’s surface and therefore dictates the strength of the Coulombic forces between the protein molecules.^6^ In addition, the pH also governs the strength of electrostatic interactions between the protein and solvent molecules as well as additives and ions present in the solution. A good example is the relationship between the isoelectric point (pI) of the protein and the solution pH in correlation to the Hofmeister series. While the order of salts in the Hofmeister series (SO_4_^2–^ > PO_4_^2–^ > Ac^2–^ > citrate^3–^ > Cl^–^ > NO_3_^–^) is only valid for acidic proteins (pI < pH 7, e.g., collagenase) that are crystallized at pH greater than the pI (pH > pI), for basic proteins (pI > pH 7, e.g., lysozyme), the Hofmeister series follows a reversed order when the crystallization pH is below the protein’s pI (pH < pI).^24^ Statistical analysis of nearly ten thousand unique protein crystal forms has revealed that for basic (acidic) proteins, a buffer pH below (above) the pI has the highest likelihood for successful crystallization and vice versa.^25,26^ Additionally, investigation of the nucleation and growth rate of the basic protein lysozyme (pI = 10.7) or the acidic protein insulin (pI = 5.3) has revealed that a shift toward a more acidic (for lysozyme) or basic (for insulin) solution pH enhances the nucleation and growth rate.^7,11,20,27,28^ Recently, it has been shown that insulin crystallization can be enhanced significantly with the addition of the basic amino acid arginine.^20^ The deprotonation of arginine leads to a pH shift toward a more basic pH, thereby, significantly enhancing the crystallization of insulin.

Thus far, limited work has been done in investigating the role of pH on human insulin crystallization, for example, only on the crystallography scale (∼μL) and only with the interference of other changing properties, such as salt type or co-solvent.^11,25,28−30^ However, no previous experimental study has investigated the influence of only the pH at scales of milliliters and without interference of other changing properties in order to derive the crystallization kinetics. In this study, crystallization of insulin over a range of pH from 6.0 to 6.7 and supersaturation ratios of up to 20.9 was carried out. Human insulin was selected because its crystallization is induced by a pH change and therefore it is extremely pH-sensitive. In addition to evaluating the crystallization kinetics as a function of pH, we investigated the pH dependence of the protein–protein interactions by measuring the second virial coefficient and hydrodynamic radius.

## Experimental Section

### Materials

All materials were used as received from the supplier without further purification or treatment unless otherwise stated. Human insulin stock solution (10.8 mg·mL^–1^ in 25 mM HEPES buffer, sterile-filtered, BioXtra, CAS number: I9278), zinc–sulfate (>99% purity), zinc chloride (>98% purity), zinc acetate (>99% purity), citric acid buffer components, and methylene blue solution were purchased from Sigma-Aldrich (UK). Eppendorf tubes (polypropylene) were purchased from FischerScientific (UK).

### Insulin Crystallization

Human insulin crystallization was carried out at volumes of 1.5 mL in Eppendorf tubes under static conditions at a constant temperature of 24.0 ± 0.1 °C. Precise temperature control was achieved by using incubators held at a constant temperature. Citric acid buffer (0.1 M) was prepared at different pH levels by varying the ratio between citric acid and tri-sodium citrate. The pH was determined using a Jenway 4330 pH and conductivity meter (Jenway, UK) with an instrument resolution of around ±0.02 pH units. Zinc sulfate (ZnSO_4_), zinc chloride (ZnCl_2_), or zinc acetate (ZnAc) were dissolved in citric acid buffer as a zinc salt stock solution. Because the purchased human insulin (CAS: I9278) is dissolved in 25 mM HEPES buffer, the amount of 25 mM HEPES buffer added was kept constant. To achieve a higher insulin concentration in the stock solution, the insulin solution was concentrated using an Amicon Ultrafiltration Unit with 3000 Da cut-off membranes (Merck, Germany). The zinc concentration in the final crystallization cocktail was adapted to the initial insulin concentration according to the molar ratio of the insulin monomer to Zn^2+^: *c̃*_insulin monomer_/*c~*_Zn^2+^_ = 0.137 because it is beneficial to use more zinc than the stoichiometric ratio.^31^ The final crystallization cocktail was achieved by mixing citric acid buffer, zinc salt stock solution, insulin solution, and HEPES buffer, which was then filtered with a 0.22 μm pore filter (PTFE membrane syringe filters, VWR, UK) immediately after mixing. The crystallization cocktail always has the following composition: 73 mM citric buffer, 7 mM HEPES buffer, 1 mM to 8.2 mM Zn^2+^, and 0.80 mg·mL^–1^ (0.14 mM) to 7.02 mg mL^–1^ (1.21 mM) human insulin. A detailed summary of the experimental condition probed is given in Table 1. Each experimental condition was repeated multiple times, and the error bars represent the deviation between these repetitions. The desupersaturation (crystallization occurs and the solution concentration decreases) was monitored by measuring the UV–vis absorption at a wavelength of 280 nm with a NanoDrop One^c^ microvolume UV–vis spectrophotometer (Thermo Fischer Scientific, USA). A sample as small as 10–20 μL was withdrawn from the crystallizer (Eppendorf tube) and was centrifuged (6000 rpm, 10–20 min). The supernatant (∼10 μL) was further diluted, and the insulin concentration of the diluted supernatant was measured with UV–vis absorption. An extinction coefficient of 1.04 mL·mg^–1^·cm^–1^ was utilized.^32^ Multiple dilutions for every sample were made to minimize dilution errors. The crystal slurry which remained in the centrifuged sample was observed underneath an optical microscope (CX-41, Olympus, Japan) to confirm that the decrease in concentration is because of crystallization and not due to other phenomena, such as amorphous precipitation.

**Table 1:** Summary of Experimental Conditions Probed for Insulin Crystallization

pH^a^	c_ins,t=0_ / mg·mL^–1^	*S*_ins,t=0_^b^/ –	zinc salt utilized	*c̃*_Zn^2+^,*t*=0_ / mM
6.0	0.80–3.01	5.6–20.9	ZnSO_4_	1.0–3.5
6.2	1.08–2.99	5.7–15.8	ZnSO_4_	1.3–3.5
6.5	2.28–6.49	5.6–15.9	ZnSO_4_	2.7–7.6
6.7	3.02–7.02	4.2–10.0	ZnSO_4_, ZnCl_2_ or ZnAc	3.5–8.2

a The pH of the crystallization cocktail after mixing all solutions.

b Supersaturation was calculated based on the solubility shown in Figure 3.

### Crystallization Data Analysis: Induction Time, Solubility, and Crystal Yield

After supersaturation was achieved at time zero, a period of time usually elapses until a substantial desupersaturation occurs. This lag time is commonly termed as induction time.^33^ We found the induction time graphically by finding the intersection between the tangents of the point of inflection (tangent of the biggest gradient after the solution concentration dropped substantially) and the initial concentration (which stays constant until the solution concentration drops substantially). A graphical visualization and a detailed definition and justification of induction time can be found in the Supporting Information. As insulin crystallizes, the solution desupersaturates and eventually reaches its equilibrium with the solid (crystal) phase. The concentration of insulin when the equilibrium was reached is the equilibrium concentration, also known as solubility c* (eq 1)
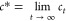
1

To investigate the impact of the solution pH, insulin crystallization was carried out under static conditions. However, mass transport of insulin molecules is very limited due to the small diffusion coefficient. To ensure that the true solubility concentration was measured, the samples were gently mixed frequently with pipettes once the crystallization experiment had finished. The crystallization experiment was assumed to be finished if the insulin concentration did not change significantly within a minimum of 12.0 h. Once the concentration had stabilized, that is, no significant change over multiple days, this concentration was taken as solubility. To ensure that the solubility was reached, the concentration was monitored over a time of 4 weeks. Within 4 weeks all samples reached their equilibrium.

The crystal yield was calculated to evaluate the performance of crystallization. The crystal yield is defined as the percentage of insulin crystalized with respect to the initial supersaturation ratio (eq 2). The yield can be either expressed in terms of concentration or supersaturation ratio, which is *S*_*t*_ = *c*_*t*_/c* for an ideal solution with *S*_*t*=0_ at time zero.
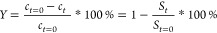
2

Where, *c*_*t*=0_ is the initial insulin concentration at *t* = 0 and *c*_*t*_ is the insulin concentration at *t* > 0. The maximum yield achievable is , that is, the yield obtained when the solid and liquid phase reached their equilibrium (c*).

### Dynamic and Static Light Scattering

Dynamic light scattering (DLS) was carried out to measure the diffusion coefficients and the hydrodynamic radii of insulin in citric acid buffer at different pHs, insulin concentrations, and different zinc salts. The solutions were prepared identically to the crystallization solutions which have been described previously. The solutions were filtered with a 0.22 μm pore size filter into a disposal cuvette (polystyrene) which then was loaded into a Zetasizer μV (Malvern, UK). The measurement was taken immediately after filtration to avoid measuring insulin crystals which will form over time. The polydispersity index was lower than 0.10 which indicates reliable measurements.

The second osmotic virial coefficient (B_22_), as a measurement of all possible pair interaction forces of insulin, was obtained by static light scattering which was carried out with a Litesizer 500 (Anton Paar, Austria). The procedure was identical to the DLS experiments, but instead of disposable cuvettes, a quartz cuvette was utilized. After each run, the quartz cuvette was carefully washed with deionized water, ethanol, and IPA followed by drying to remove any impurities and dust before further usage for the next sample. A solvent refractive index of 1.3304 and a refractive index increment of Δ*n*/Δ*c* = 0.183 were utilized.^34^ The second virial coefficient was obtained by using Debye plots at low protein concentration (≤ 3 mg·mL^–1^). The median light scattering intensity (*Kc*/*R*_θ_) is plotted over the insulin concentration, and with eq 3, the B_22_ (slope of linear fit) and molecular weight (1/intercept) are derived.
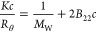
3where *K* is a system constant, *R*_θ_ is the Rayleigh ratio, *c* is the protein concentration, and M_W_ is the molecular weight.

## Results

### Desupersaturation of Insulin

**Figure 1:**
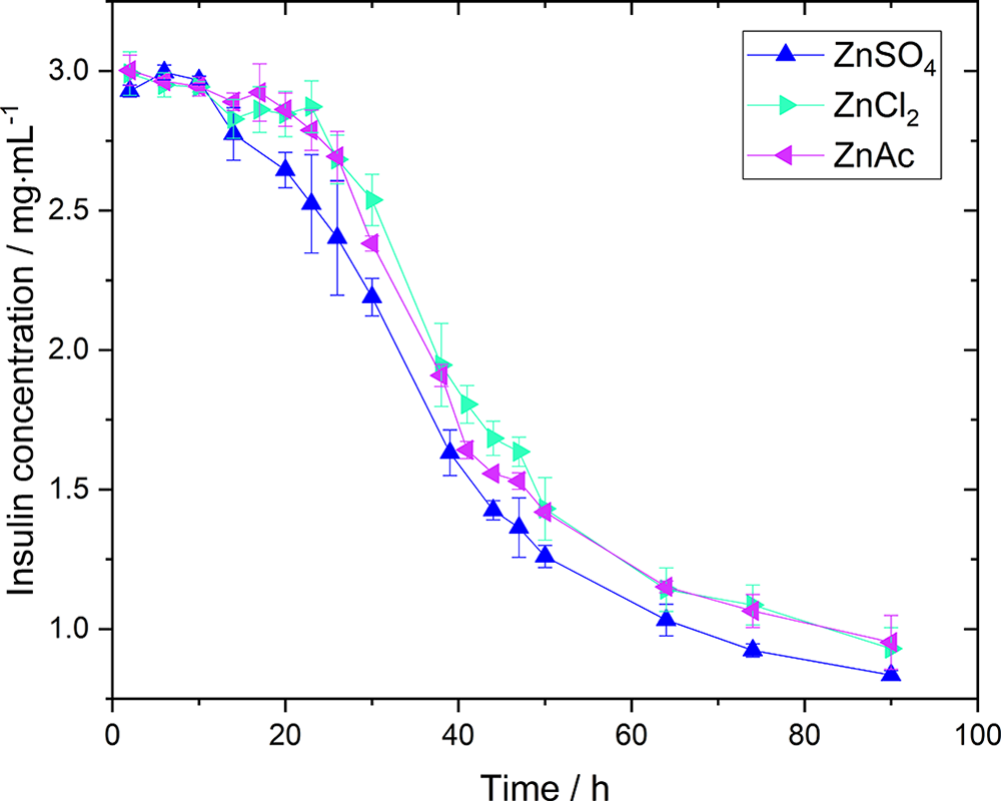
Desupersaturation curve for insulin in the presence of different zinc salts with concentrations of 3.5 mM at pH 6.7 and 24.0 °C.

To form the rhombohedral crystal shape, insulin must be assembled in its hexametric form which can be achieved with the addition of zinc salts. Thereby, two Zn^2+^ ions coordinate the His10 residues of three insulin dimers to form the hexametric unit. Here, we investigated the impact of different zinc anions (Cl^–^, SO_4_^2–^, or Ac^2–^) at a fixed concentration of 3.5 mM on the crystallization of insulin to screen for the most promising salt in enhancing crystallization (Figure 1). While the addition of salts did not change the pH nor the solubility (see Table 2), the fastest desupersaturation was observed when ZnSO_4_ is added compared to ZnCl_2_ or ZnAc while there is no significant difference in desupersaturation between the latter two (Figure 1). Adding ZnSO_4_ results in the shortest induction time (19.5 ± 2.0 h) compared to the addition of ZnCl_2_ (23.5 ± 0.5 h) or ZnAc (23.0 ± 0.5 h).

**Table 2:** Solubility and Diffusion Coefficient of Insulin as a Function of the Zinc Anion Type at 24.0 °C^a^

anion	pH / –	insulin equilibrium concentration / mg·mL^–1^	diffusion coefficient / 10^–6^ cm^2^·s^–1^
SO_4_^2–^	6.7	0.70 ± 0.03	0.86 ± 0.8·10^–3^
Cl^–^	6.7	0.67 ± 0.09	0.87 ± 0.5·10^–3^
Ac^2–^	6.7	0.67 ± 0.04	0.87 ± 0.1·10^–3^

a The diffusion coefficient was measured at *c*_ins_ = 3 mg·mL^–1^.

After nucleation has occurred, it seems that the zinc salt does not have any impact on the crystallization rate, that is, the change in supersaturation or yield over time (e.g., d*S*/d*t* or d*Y*/d*t*), as the change in insulin desupersaturation after roughly 35.0 h is similar. For the addition of ZnSO_4_, the insulin crystallization rate is 0.06 h^–1^ [1.63 mg·mL^–1^ at 39.0 h (*S*_*t*=39h_ = 2.3) to 1.43 mg·mL^–1^ at 44.0 h (*S*_*t*=44h_ = 2.0)], while for ZnCl_2_, the crystallization rate is 0.07 h^–1^ [1.68 mg·mL^–1^ at 44.0 h (*S*_*t*=44h_ = 2.5) to 1.43 mg·mL^–1^ at 50.0 h (*S*_*t*=50h_ = 2.1)]. Similar to the crystallization rates, no differences in growth rates were observed (d*S*/d*t* is similar for all salts between 74.0 and 90.0 h).

**Figure 2:**
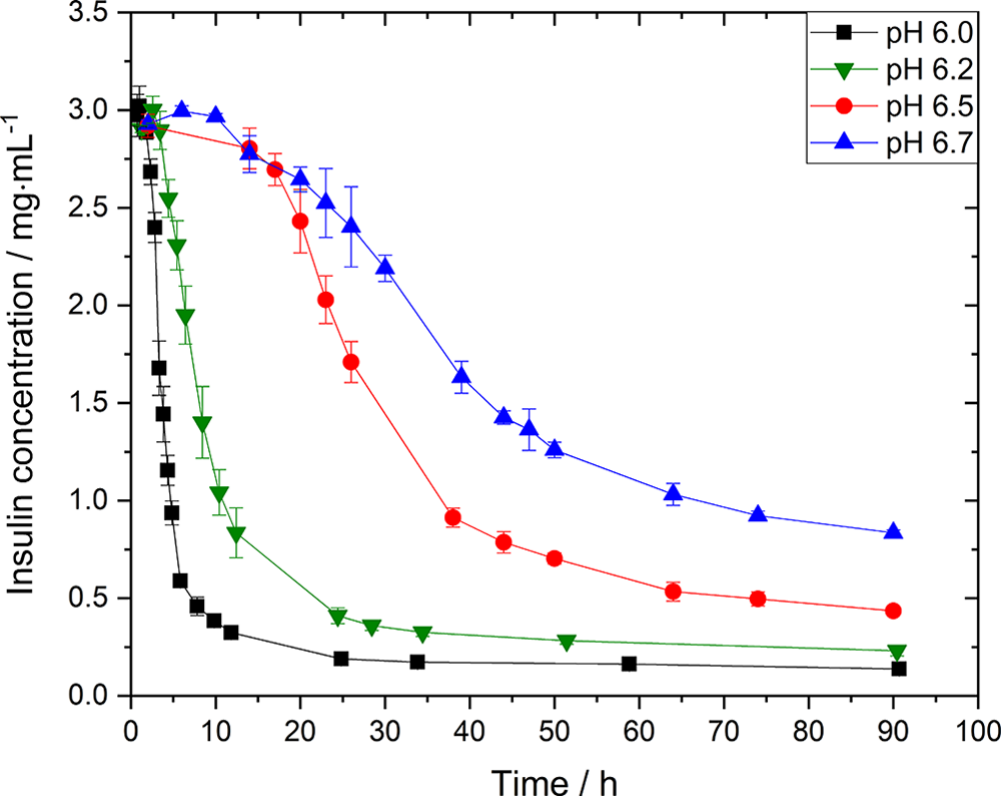
Insulin desupersaturation curves at different pH with an initial insulin concentration of 3 mg·mL^–1^ (*S*_*t*=0_ = 20.9 at pH 6.0 to *S*_*t*=0_ = 4.2 at pH 6.7) and a ZnSO_4_ concentration of 3.5 mM at 24.0 °C.

As ZnSO_4_ led to the fastest desupersaturation, it was utilized for the further investigation of pH on insulin crystallization. To obtain the induction time, crystal yield, and solubility as a function of pH, the desupersaturation was measured over time at different pH with the addition of ZnSO_4_ (Figure 2). As described in the methods, the Zn^2+^ concentration was adjusted to the insulin concentration. A decrease in pH of less than one unit (pH 6.7 to 6.0), by keeping the initial insulin concentration constant, results in a faster desupersaturation, representing a faster crystallization, as the concentration drops after 4.0 h at pH 6.0 compared to 19.5 h at pH 6.7 and a quasi-equilibrium concentration can be reached four times faster (roughly 20.0 h at pH 6.0 compared to 80.0 h at pH 6.7). The achieved equilibrium concentration, also known as equilibrium solubility, decreases with decreasing pH. To confirm the occurrence of crystallization and not any other forms of precipitation, we monitored the supernatant after centrifugation with an optical microscope and observed well-built rhombohedral insulin crystals (see Supporting Information Figure S1).

### Insulin Solubility as a Function of pH

**Figure 3:**
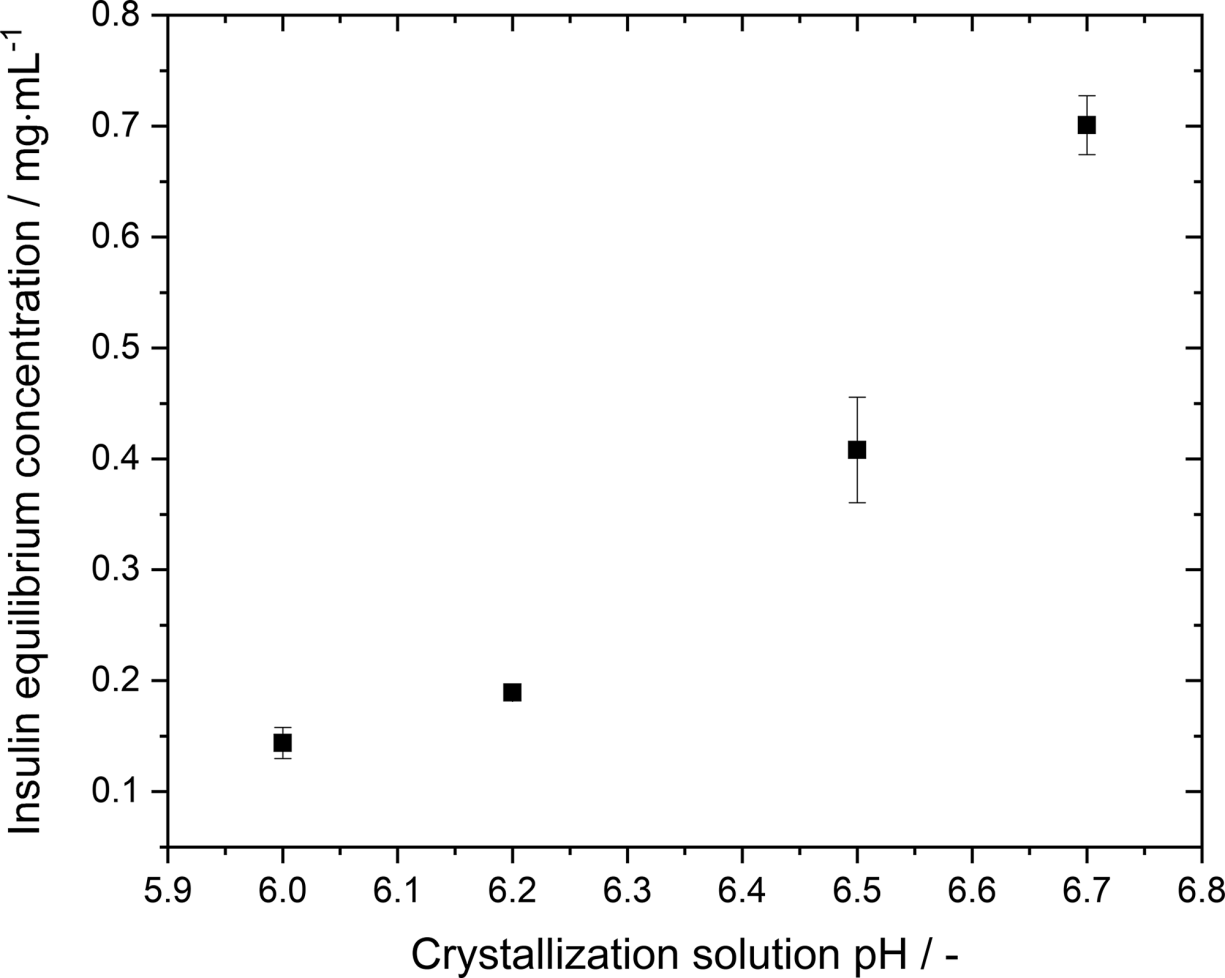
Insulin solubility as a function of pH with the addition of ZnSO_4_ at 24.0 °C.

From Figure 2, the insulin solubilities at different pHs can be derived (see Figure 3). Measuring the concentration periodically after crystallization has finished, it was found that after 2–3 weeks the concentration did not change anymore. Additionally, the pH was measured and in the very rare case of a significant change in pH, <1% off all experiments, these experiments were consequently discarded. The solubility of insulin increases with increasing pH from 0.14 mg·mL^–1^ at a pH of 6.0 to 0.70 mg·mL^–1^ at a pH of 6.7 which is a 5-fold increase in solubility by a pH change of less than one pH unit. The solubility follows a nonlinear trend and highlights that a small pH change such as 0.2 units can increase the solubility by roughly 70% (*c*_pH=6.5_^*^ = 0.41 mg·mL^–1^ → *c*_pH=6.7_^*^ = 0.70 mg·mL^–1^). Our obtained solubility values are in good agreement with solubility concentrations reported in the literature (0.15 to 0.18 mg·mL^–1^ at a pH around 6.2^35−38^). We observed a nonlinear trend of the increase in insulin solubility with increasing pH which is as expected as the solubility of insulin is the lowest around its pI, which is around 5.3 for human insulin and increases with increasing or decreasing pH. This nonlinear correlation between pH and solubility is corroborated by solubility studies of other proteins.^4,39,40^ We also monitored the solution over time and always observed crystals, which confirms that insulin is stable in the utilized condition.

### Impact of Supersaturation and pH on the Crystallization Rate of Insulin

**Figure 4:**
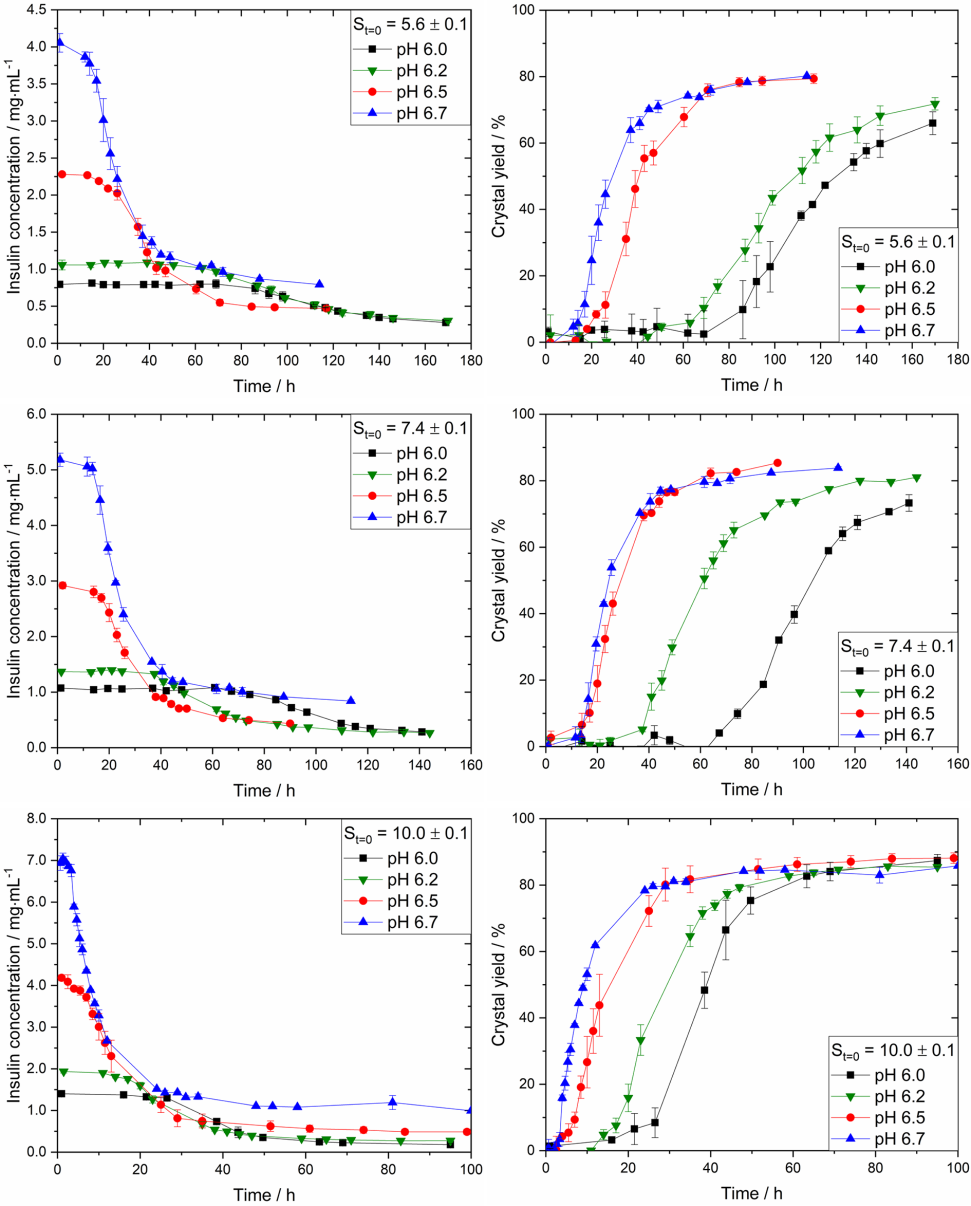
Desupersaturation curve (left) and obtained crystal yield (right) for insulin crystallization at various supersaturation ratios and pH at 24.0 °C. The average pH values are 6.0, 6.2, 6.5, and 6.7.

To evaluate the impact of pH and consequently the net surface charge on insulin crystallization, crystallization experiments at different pHs and initial supersaturation ratios (*S*_*t*=0_) were carried out. Figure 4 displays the desupersaturation (left) and crystal yield (right) over time for insulin at three distinct initial supersaturation ratios (*S*_*t*=0_ = 5.6 ± 0.1, 7.4 ± 0.1, and 10.0 ± 0.1) and four solution pHs (pH = 6.0, 6.2, 6.5, and 6.7). Keeping the pH constant and increasing the initial supersaturation, the insulin concentration decreases faster over time, resulting in a steeper desupersaturation, and therefore an increased crystallization rate, that is, the change in supersaturation or yield over time (e.g., d*S*/d*t* or d*Y*/d*t*). For example, at pH 6.7 and *S*_*t*=0_ = 5.7 the concentration decreases from 3.90 mg·mL^–1^ at 12.0 h (*S*_*t*=12h_ = 5.6) to 1.36 mg·mL^–1^ at 41.0 h (*S*_*t*=41h_ = 1.9) resulting in d*S*/d*t* ≈ Δ*S*/Δ*t* = 0.13 h^–1^. At pH 6.7 and *S*_*t*=0_ = 10.0, the concentration decreases from 6.90 mg·mL^–1^ at 1.3 h (*S*_*t*=1.3h_ = 9.8) to 1.37 mg·mL^–1^ at 26.0 h (*S*_*t*=26h_ = 2.0) resulting in Δ*S*/Δ*t* = 0.32 h^–1^. The same trend can be observed for the crystallization rates at the other pH values. On the other hand, the crystallization rate increases with increasing pH at a constant supersaturation ratio. For instance, at *S*_*t*=0_ = 10.0, the crystallization rate increases from 0.18 to 0.32 h^–1^ with an increase in pH from 6.0 to 6.7. Additional calculations and values of the desupersaturation rates (Δ*S*/Δ*t*) can be found in the Supporting Information.

Because the initial supersaturation for different pH was kept constant, the maximum yield achievable (Y*) is independent of the pH. First, with increased supersaturation the *t*_50%_ (time required to achieve a yield of 50%) decreases from 128.0 to 39.0 h (pH 6.0) and from 31.0 to 3.5 h (pH 6.7) when the initial supersaturation ratio is increased from 5.6 to 10.0 (see Supporting Information, Figure 3). This is expected because a higher initial supersaturation leads to faster desupersaturation due to the increased nucleation rate and faster crystal growth rate. On the other hand, the *t*_50%_ decreases from 128.0 to 31.0 h (*S*_*t*=0_ = 5.6) and from 39.0 to 3.5 h (*S*_*t*=0_ = 10) if the pH is increased (6.0 → 6.7) and the initial supersaturation ratios are kept constant at 5.6 and 10.0 respectively.

**Figure 5:**
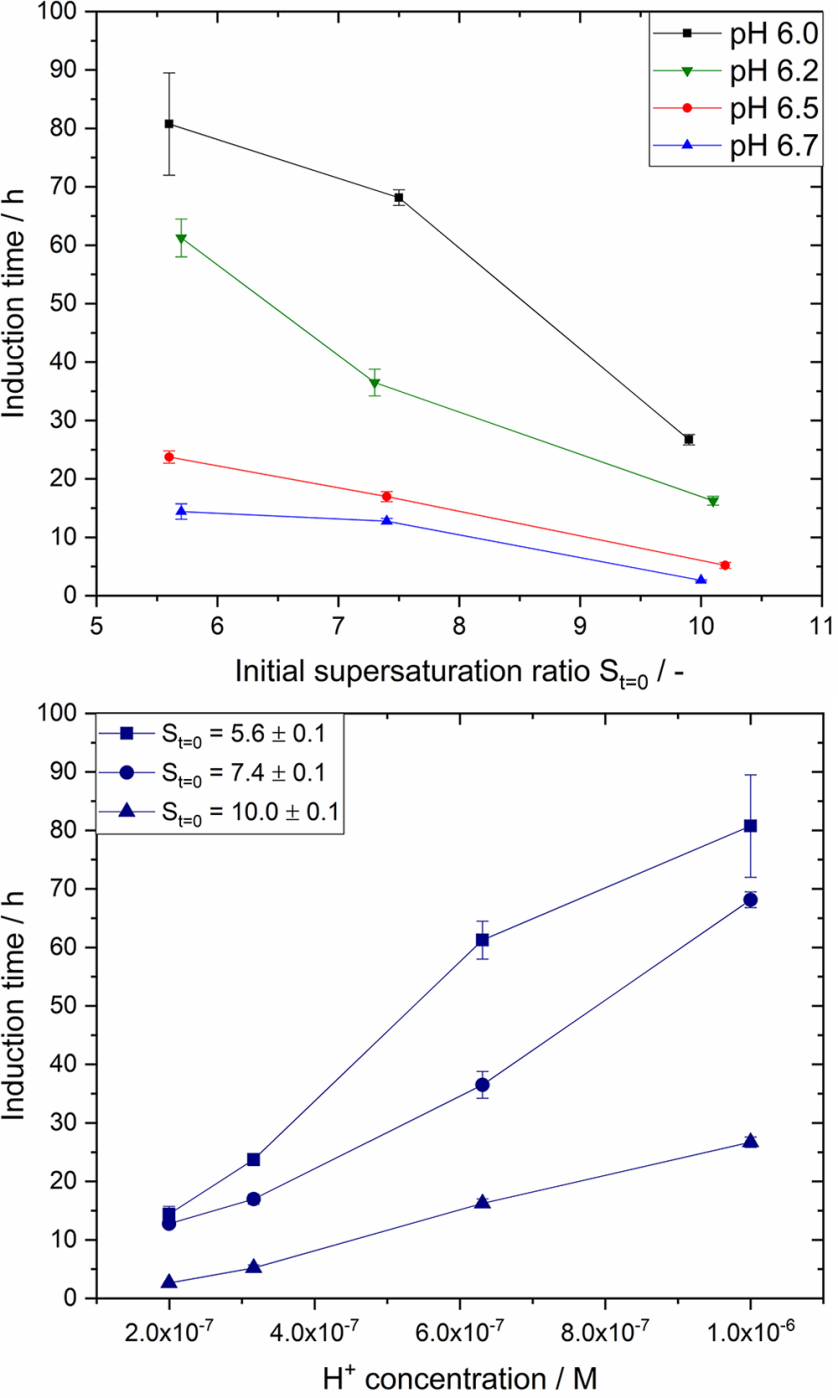
Induction time of insulin crystallization as a function of the initial supersaturation ratio at constant pH (top) and as a function of H^+^ concentration in solution at a constant initial supersaturation ratio (bottom) at 24.0 °C.

Besides investigating the impact of solution pH and initial supersaturation ratio on crystal yield and desupersaturation rate, the impact on induction time was also evaluated. Here, we define induction time as the time period until a substantial decrease in insulin concentration was observed (see Supporting Information for detailed explanation). Figure 5 shows the obtained induction times as a function of initial supersaturation or H^+^ concentration. At a constant pH, the induction time decreases with increasing supersaturation. An increase in the initial supersaturation ratio from 5.6 to 10.0 results in a reduction in induction time by 67% (e.g., 81.0 to 26.5 h), 75, 77, and 79% for pH 6.0, 6.2, 6.5, and 6.7, respectively. It seems that at a more basic pH the magnitude in induction time reduction is greater (e.g., 67% at pH 6.0 compared to 79% at pH 6.7). From the pH the H^+^ concentration in the solution can be calculated with *c̃*_H^+^_ = 10^–pH^, assuming that the solution behaves as an ideal solution. The induction time decreases with decreasing H^+^ concentration at a constant initial supersaturation ratio (Figure 5), meaning a more basic milieu is more beneficial for insulin crystallization. Reducing the H^+^ concentration in solution from 1.0 × 10^–6^ M (pH 6.0) to 2.0 × 10^–7^ M (pH 6.7) results in a decrease in induction time by roughly 81% at *S*_*t*=0_ = 7.4 and 88% at *S*_*t*=0_ = 10.0. It seems that the relationship between induction time and H^+^ concentration is linear.

A low supersaturation is required for solely crystal growth to occur. A crystal yield of 85% represents a supersaturation ratio of 1.5 and is achieved after 87.0, 83.0, 61.0, and 58.0 h for *S*_*t*=0_ = 10.0 at pH of 6.0, 6.2, 6.5, and 6.7, respectively. The desupersaturation rates after a yield of 85% was achieved were 0.03, 0.01, 0.07, and 0.02 h^–1^. It seems that the rate at which the concentration decreases and, hence, the yield increases, is independent of the pH and the initial supersaturation. The similar rates in desupersaturation over time indicate that the growth rate is independent of the pH.

**Figure 6:**
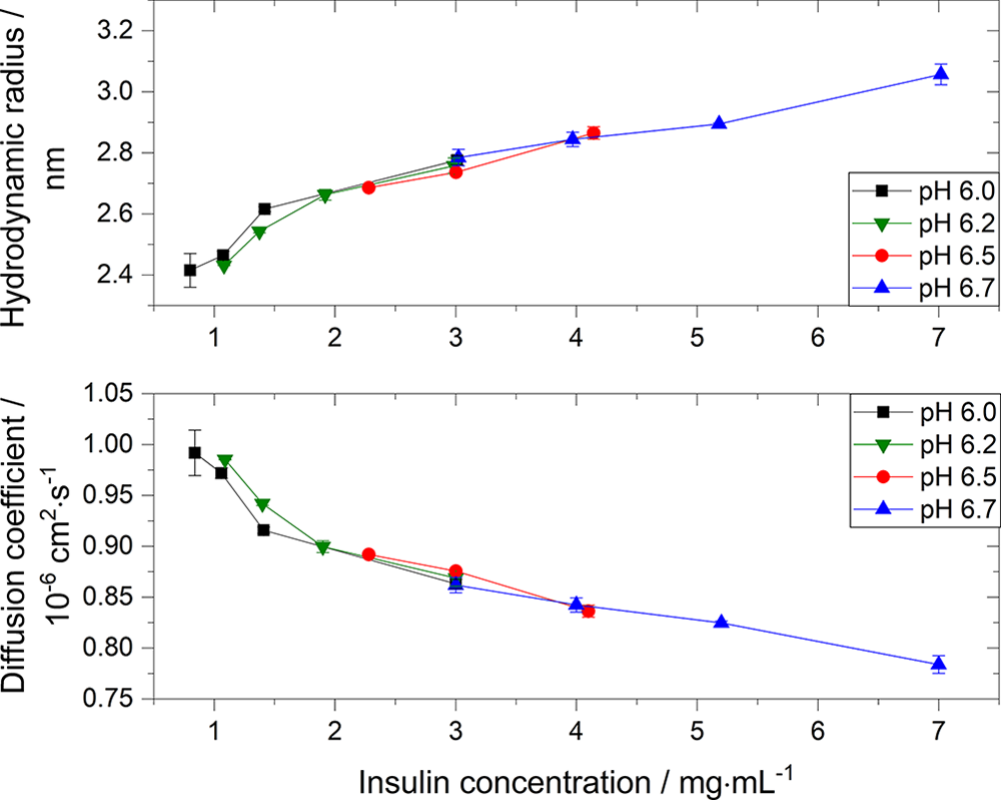
Hydrodynamic radius and diffusion coefficient of insulin at different solution pHs as a function of concentration at 24.0 °C.

To shed light on the mass transfer under static conditions, the diffusion coefficient (*D*) of insulin was measured (Figure 6). The diffusion coefficient seems to be independent of the solution pH and anion type (Table 2) and overall decreases with increasing insulin concentration (e.g., from 0.99 × 10^–6^ to 0.86 × 10^–6^ cm^2^·s^–1^ at a pH of 6.0). The decreasing diffusion coefficient with increasing protein concentration and the independence of the pH agrees with diffusion coefficient studies of hen egg-white lysozyme.^26^ While the diffusion coefficient is directly derived from the correlation function, the hydrodynamic radius (*R*_H_) is calculated from the diffusion coefficient with the Stokes–Einstein equation (*R*_H_ ∝ *D*^–1^). Hence, a decrease in diffusion coefficient results in an increase in hydrodynamic radius (Figure 6). We studied the hydrodynamic radius over time prior to crystallization to confirm that insulin is solely in its hexametric form prior to crystallization throughout all experiments, as we only observed a monomodal intensity distribution with a polydispersity index of less than 0.10. This also confirms that no aggregation occurs during the DLS measurements.^31,41^

## Discussion

### Role of Anions in the Crystallization of Insulin

**Figure 7:**
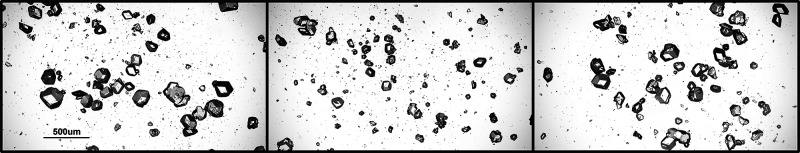
Insulin crystals with the addition of ZnCl_2_ (left), ZnAc (middle), or ZnSO_4_ (right) at 24.0 °C. Images were taken after 90.0 h. Scale bar is for all images.

It is well known that metal ions are required to form the more stable and crystallizable insulin hexamer.^42−44^ Here, we studied the impact of different types of zinc salt on the crystallization kinetics of human insulin. Zinc chloride, zinc sulfate, or zinc acetate are commonly used as zinc-providing salts and have therefore been selected.^11,20,45^ As the addition of ZnSO_4_ led to the shortest onset of nucleation compared to the addition of ZnCl_2_ or ZnAc it seems that insulin crystallization with the addition of SO_4_^2–^, Ac^2–^ or Cl^–^ follows the Hofmeister series, that is SO_4_^2–^ > PO_4_^2–^ > Ac^2–^ > citrate^3–^ > Cl^–^ > NO^3–^ for negatively charged proteins.^13^ Because the insulin solubility is independent of the zinc salt type added, we hypothesize that SO_4_^2–^ is more kosmotropic and, hence, promotes salting-out compared to Ac^2–^ or Cl^–^. All three zinc salts lead to the formation of the rhombohedral crystal shape, and no change in size was observed (see Figure 7). Additionally, it has been shown that the accommodation of insulin hexamers within the crystal differs between a variety of conformations, such as T_6_, T_3_R_3,_ and R_6_. The R_6_ conformation is obtained if phenol and Cl^–^ (e.g., in the form of ZnCl_2_) are added, the T_3_R_3_ is obtained if only Cl^–^ is added and the T_6_ conformation is obtained if neither phenol nor chloride is added (e.g., ZnSO_4_).^44,46−48^ While the chloride anions are coordinated around the Zn^2+^ ions and HisB10 residues in the crystal lattice, the sulfate anions are not incorporated into the crystal resulting in R_6_ and T_3_R_3_ being more compact than the T_6_ conformation.^49^ The less compact crystal configuration with the addition of SO_4_ could be a result of a faster nucleation. Ultimately, we conclude that the choice of the anion helps promote insulin crystallization as we found that SO_4_^2–^ is a stronger kosmotropic salt compared to Cl^–^ or Ac^2–^. The impact of the anion on crystallization is supported by studies on the crystal conformation as Cl^–^ or SO_4_^2–^ lead to different crystal conformations.

### Insulin Solubility

**Figure 8:**
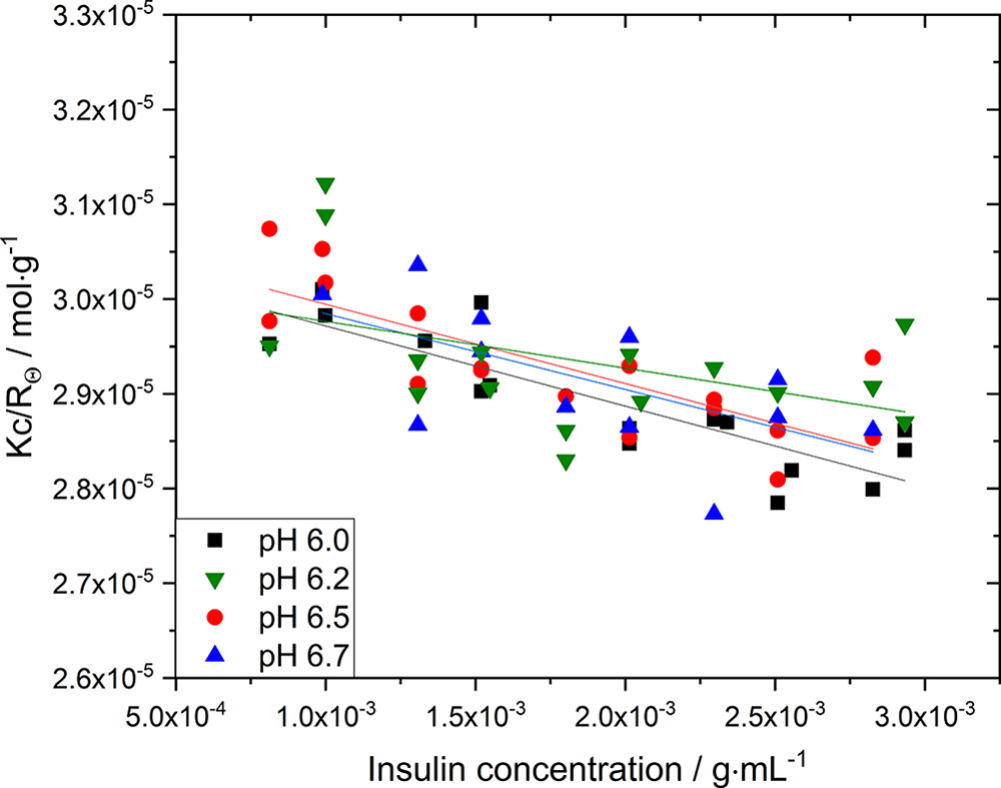
Intensity of scattered light (*Kc*/*R*_θ_) as a function of insulin concentration for different solution pH at 24.0 °C. An average molecular weight of 32.7 kDa for the insulin hexamer was found.

By increasing solution pH (pH 6.0 → 6.7), the net surface charge of insulin becomes more negative due to deprotonation of some of the residues as the pI of insulin is around pH 5.3. It is well known that at low solubilities, an increase in solubility correlates with an increase in repulsive electrostatic protein–protein interactions.^17,50,51^ However, this contradicts our initial findings on the hydrodynamic radius which is independent of the solution pH (see Figure 6) at insulin concentration of 3 mg·mL^–1^ and hence indicates that the change in solubility is not correlated to a change in protein–protein interactions. To prove this, we experimentally determined the second virial coefficient (B_22_) at different solution pH (see Figure 8 and Table 3). The obtained B_22_ seems to be independent of the pH as, with the exemption of pH 6.2, the B_22_ increases by 0.2·10^–4^·mL·mol·g^–2^ from pH 6.0–6.7. Although it has been shown that even a small change in B_22_ (ΔB_22_ = 1.0·10^–4^·mL·mol·g^–2^) could lead to a two-fold increase in solubility,^50−53^ our findings show a solubility increase of 5 folds while the B_22_ changes by 0.2·10^–4^ mL·mol·g^–2^ from pH 6.0 to 6.7. Due to the small change in B_22_ and the significant change in solubility, we suspect that the increase in solubility is due to a stronger and further-reaching hydrogen network between the negatively charged insulin hexamers and the water molecules in the hydration shell resulting in stronger solvation. Recent research on the impact of electrostatic interaction between proteins supports our hypothesis.^6,17^

**Table 3:** Dependency of the Second Virial Coefficient, Obtained from Figure 8, and the Derived Activity Coefficient of Insulin of Solution pH at 24.0 °C

pH / –	B_22_ / mL·mol·g^–2^	γ / –
6.0	–4.23 × 10^–4^	0.99
6.2	–2.47 × 10^–4^	0.99
6.5	–4.18 × 10^–4^	0.98
6.7	–4.0 × 10^–4^	0.96

To justify the assumption of an ideal equilibrium between the crystals and the solution, we calculated the activity coefficient (*γ*) from the B_22_ with eq 4,^50^ where *M*_Insulin_ is the molecular weight of an insulin hexamer because, in the presence of Zn^2+^ ions, insulin is solely present in its hexametric form (see results on hydrodynamic radius). From the Debye plot, we obtained an insulin hexamer molecular weight of 32.7 kDa, which is close to the reported value of 34.9 kDa for recombinant human insulin.
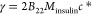
4

The lowest obtained activity coefficient is 0.96 for the highest pH 6.7 (see Table 3), which is ≈1 and hence justifies the assumption of an ideal solution (*S* = *c*/c*).

### Impact of pH on Induction Time and Crystallization Rate

We found that with increasing supersaturation ratio at a constant pH or with increasing pH at a constant supersaturation ratio, the induction time decreases, representing an increase in nucleation rate (*J*) because the induction time is directly proportional to the reciprocal of the nucleation rate (*J* ∝ *t*_ind_^–1^).^33^ The nucleation rate as a function of supersaturation for an ideal solution can be expressed with the classical nucleation theory according to Mullin, Vekilov, and Schall (see eq 5).^22,33,54^ However, it must be stated that detecting the onset of nucleation is challenging and often can be detected at considerable times after the very first nucleus appeared. Hence there is always a bias toward the parameter A and B derived from induction time measurements that stems from the resolution and accuracy of the applied analytical technique. As our objective was to obtain a relativity between the parameter A and B for homogeneous nucleation and the pH and not an absolute value, we demonstrated that (*J* ∝ *t*_ind_^–1^) is valid for the evaluation of A and B from our obtained desupersaturation curves (see Supporting Information for detailed discussion on induction time and validation). Consequently, our first observation of increased nucleation rate is due to an increased protein concentration and consequently supersaturation ratio.
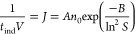
5where *t*_ind_ is the induction time, *V* is the volume of solution, *A* is a parameter describing the molecular attachment kinetics, *B* is related to the nucleation barrier which must be overcome, and *n*_0_ is the insulin hexamer number density as this is the crystallizing species. The number density is directly proportional to the concentration of insulin hexamers. An interesting point here is that the reduction in induction time with increasing initial supersaturation also increases with increasing pH (see the shift in induction time decrease from 67 to 79% if the pH is increased from 6.0 to 6.7). This indicates increased facilitation in crystallization when a more basic pH is chosen for an acidic protein such as human insulin.

**Figure 9:**
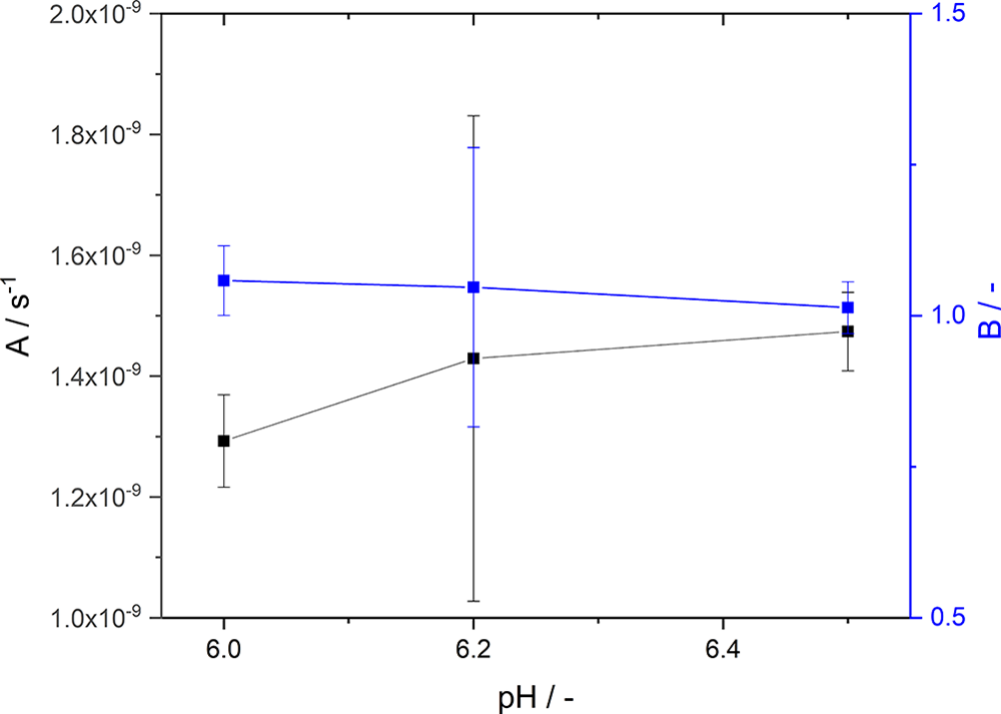
Estimated parameter A and B of the classical nucleation equation (eq 5) for homogeneous nucleation as a function of pH at 24.0 °C. For discussion, see text and derivation in Supporting Information.

Second, shifting the pH toward a basic milieu at a constant supersaturation increases the nucleation rate. To prove that a more basic pH enhances the nucleation rate, we plotted ln(*J*/*n*_0_) versus ln(*S*)^−2^ to obtain the concentration-independent values for the parameter A and B for homogeneous nucleation (see Supporting Information for detailed derivation and explanation). While B does not change with increasing pH, we found that A increases slightly by 14% from pH 6.0 to 6.5 (see Figure 9), indicating a more basic pH favors insulin crystallization which agrees with similar studies on the salting-out of lysozyme.^54^ However, the parameter A is a complex parameter that depends on the attachment and mass transport kinetics, and it has been shown that it is often not suitable to lump the kinetics in protein crystallization in one parameter.^22^ It was not possible to obtain A and B for pH 6.7, as this would require an initial insulin concentration of >11.8 mg·mL^–1^, which was not able to be achieved with centrifugation. Additionally, it must be stated that a significant contribution to the faster nucleation at higher pH is due to the higher insulin hexamer number density (insulin concentration). In addition to describing the role of pH on the nucleation behavior of insulin with a classical nucleation approach, we studied the protein–protein interactions. Our results show that the protein–protein interactions are independent of the pH, as neither R_H_ nor B_22_ changes with changing pH (see Figures 6 and 8), and consequently, the increase in nucleation rate is not due to enhanced attractive interactions. The independence of the protein–protein interactions on the pH also proves that no ionic shielding of the protein’s surface in solution occurs, as this would have resulted in a decrease in repulsive interactions (e.g., decreased B_22_ or increased R_H_), which we did not observe. Therefore, we think it is the stronger interaction between water molecules and protein surfaces in combination with ions in solution that leads to an increase in the hydrogen bond strength between the water molecules in the hydration layer and the residues on the insulin surface that ultimately results in an enhanced crystallization. It is well recognized that the electrostatic interaction between the protein’s residue and the water molecules, and the resulting water structuring around the protein’s surface, plays a major role in protein dynamics^55,56^ and crystallization.^6−8,57^ In the case of human insulin, acetone has been shown to destroy the shell of structured water around the insulin molecules. This disruption in the shell of structured water led to a 5 times greater kinetic growth coefficient.^35,36^ Hence, we attribute the faster nucleation at constant supersaturation to a change in water structuring around the insulin’s surface due to the change in net negative surface charge. For the basic protein lysozyme, a similar effect was found when the pH was lowered, from 5.2 to 4.0.^28^

The desupersaturation profile and crystal yield were chosen to evaluate the impact of the initial supersaturation and solution pH on the crystallization rate of insulin. We showed that the crystallization rate increases with increasing pH and that the same yield can be achieved within a shorter time (shifts to lower times if the pH is increased). After the first nucleation occurs, secondary nucleation will occur and will overlap with crystal growth leading to a fast desupersaturation. Hence, it is difficult to uncouple the period in which only nucleation occurs from the period in which only growth occurs. Literature suggests that a supersaturation around *S* ≈ 1.3 for insulin is low enough for just growth to occur.^58^ Comparing the change in supersaturation at the very end of the desupersaturation profile reveals that there is no difference in the crystallization rate at such low supersaturations between the pH values, indicating that the growth rate is independent of the pH. Because the nucleation rate is dependent on the solution pH, we would have expected an impact of pH also on the crystal growth rate, as computational studies suggest a pH dependency on the crystal growth rate.^7^ From this observation, in addition to the observation of a pH-independent diffusion coefficient, we hypothesize that the growth rate-limiting step is the mass transport and not the surface integration, which agrees with the suggestion that flow intensification enhances nucleation and growth.^59^

## Conclusion

This work describes a detailed investigation of the role of solution pH in combination with a variation of zinc salts on the crystallization of human insulin. We demonstrated that a more basic milieu for an acidic protein like insulin led to an increase in solubility by up to 5-fold but also to a faster onset of nucleation and increased crystallization rate. Studying the second virial coefficient as well as the hydrodynamic radius of insulin at different pHs revealed that the pH does not change the protein–protein interactions. As the net negative charge of insulin increases with an increasing pH, we hypothesize that it is the change in water structuring around the protein’s surface due to the change in electrostatic interactions between the water and protein molecules which ultimately facilitates nucleation and crystallization. Studying the role of zinc sulfate, zinc chloride, or zinc acetate on crystallization, we found that these salts follow the Hofmeister series with sulfate being the most effective anion in promoting nucleation.

In conclusion, this study gives insights into the role of pH on crystallization which will be of significant importance in designing novel crystallization pathways for the pharmaceutical industry.

## Supporting Information Available

Crystal images, derivation, definition, and justification of induction times; desupersaturation and crystallization rates as a function of pH; hydrodynamic properties of insulin in the solution studied; and derivation of the characteristic parameters for homogeneous nucleation of the classical nucleation equation (PDF)
