# Supplementary material for: Unraveling the Impact of pH on the Crystallization of Pharmaceutical Proteins: A Case Study of Human Insulin
Source: Cryst Growth Des. 2022 Apr 12;22(5):3024–33. doi: 10.1021/acs.cgd.1c01463 (PMC9073949; doi:10.1021/acs.cgd.1c01463)
Supplement: Supplementary file 1 — cg1c01463_si_001.pdf [file cg1c01463_si_001.pdf]

Unraveling the Impact of pH on the Crystallization of Pharmaceutical Proteins: A Case Study of Human Insulin

Frederik J. Link[a] and Jerry Y. Y. Heng*[a,b]

[a] Department of Chemical Engineering, Imperial College London, South Kensington Campus, London SW7 2AZ, UK

[b] Institute for Molecular Science and Engineering, Imperial College London, South Kensington Campus, London SW7 2AZ, UK

***Corresponding author:** Jerry Y. Y. Heng; jerry.heng@imperial.ac.uk

Crystal Images as Proof for Crystallization

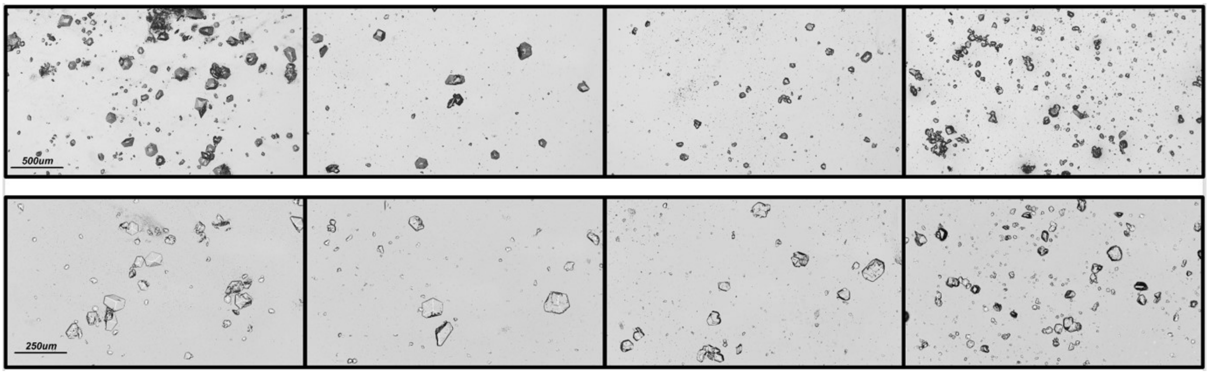

Figure 1. Images of insulin crystals obtained with an initial insulin concentration of 3 mg·mL^{-1} at different pH and 24.0 °C and correspond to Figure 2 in the manuscript; From left to right: pH 6.0, pH 6.2, pH 6.5, pH 6.7. Images shown were taken with different magnifications (top row: 5x; bottom row: 10x). The scale bar accounts for each row. It should be noted that the crystal sizes and number are not representable, and the purpose is solely for proof of crystallization.

Induction Time Determination and Desupersaturation Rates

The induction time is the time period which elapses between the achievement of supersaturation and the appearance of crystals and is made up of several parts: (1) relaxation time, (2) time required for achieving a stable nucleus and then (3) the time required for the nucleus to grow to a detectable size [1]. Thereby, the induction time depends on the resolution of the analytical technique that is utilized, and therefore, detecting the very first crystal or any change in solution is challenging and often can only be detected considerable time after the very first nucleus appeared. Hence, we are using the latent period as a mean of induction time to compare the onset of crystallization. The latent period is defined as the time which passes by until a significant change in the insulin concentration occurs which we obtain via the tangent method:

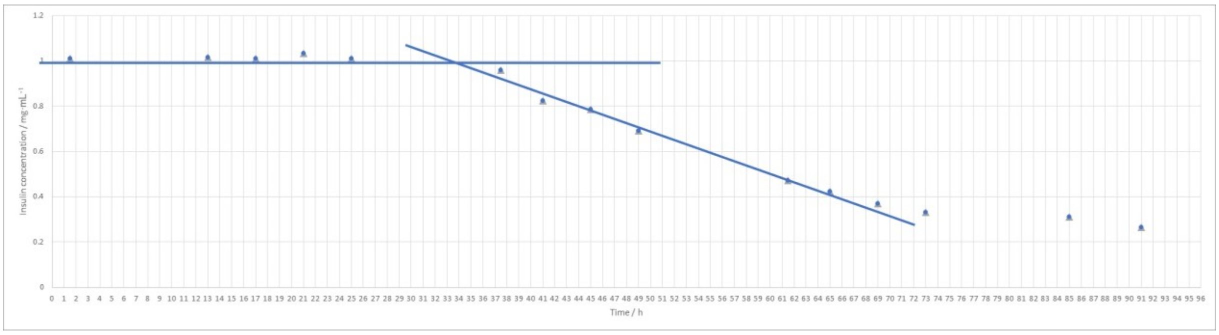

Figure 2. Schematic showing the determination of the induction time which is the interception of the two tangents. The determined induction time was 34.0 h.

In the last chapter of the Supporting Information we discuss the validation of latent period = induction time and show that this assumption holds.

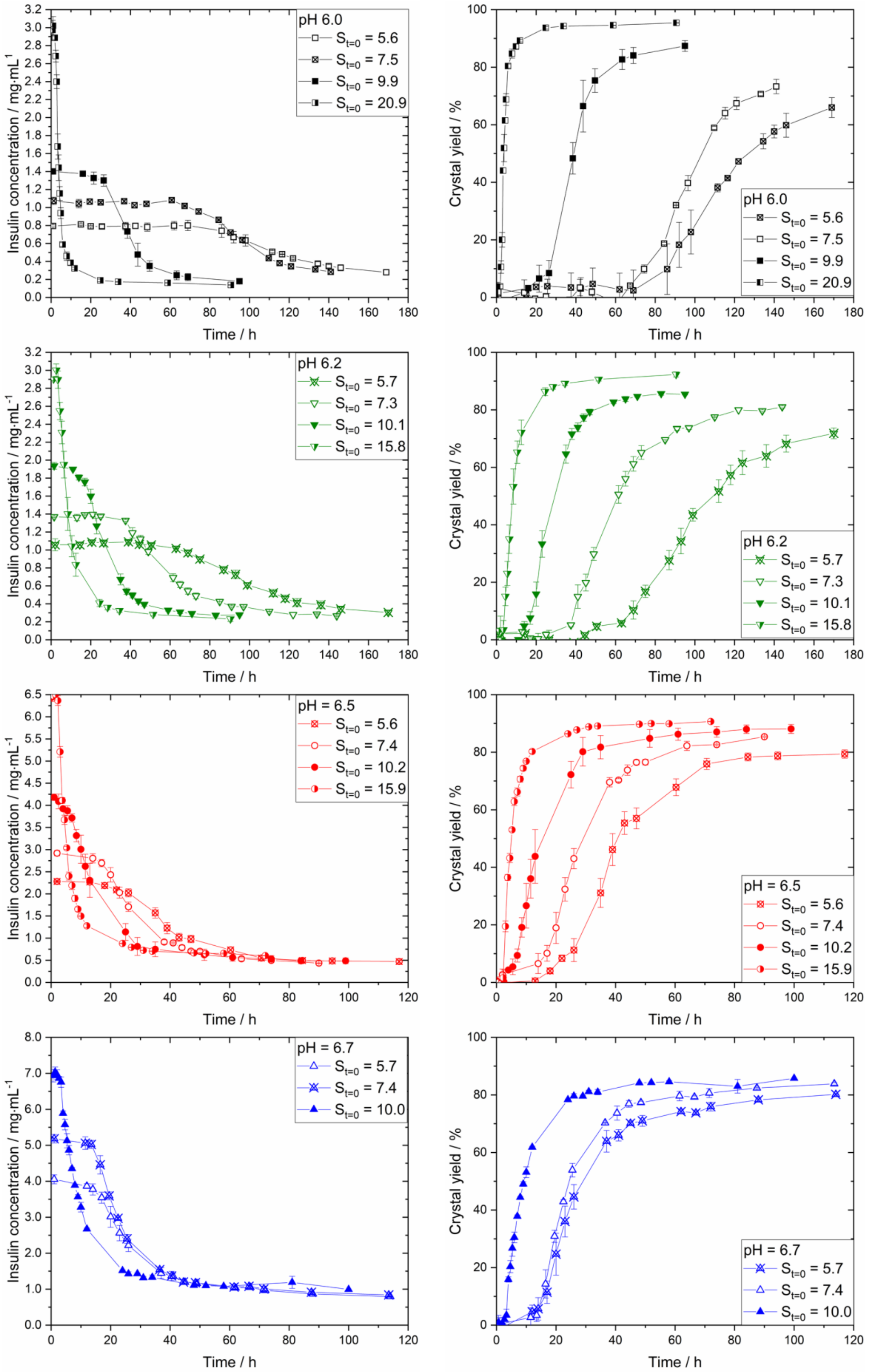

Figure 3. Desupersaturation curve (left) and obtained crystal yield (right) for insulin at various pH and supersaturation ratios at 24.0 °C.

Table 1. Crystallization rate calculation dS/dt which was numerical approximated with $\Delta S/\Delta t$. The first concentration was chosen at the time when the insulin concentration starts to decrease significantly, and the second concentration was chosen at the time when the insulin concentration approaches a plateau at which the concentration does not change significantly.

pH	$S_{t=0}$	Time / h	S_t / -	Time / h	S_t / -	${dS}/{dt} = {\Delta S}/{\Delta t}$ / $\frac{1}{h}$
6.0	5.6	69.0	5.5	169.0	1.9	0.04
	9.9	21.5	9.2	63.3	1.7	0.18
6.2	5.7	63.0	5.4	146	1.8	0.04
	10.1	11.0	10.2	47.0	2.1	0.23
6.5	5.6	13.0	5.6	60.3	1.8	0.08
	10.2	4.0	9.6	29.0	2.0	0.30
6.7	5.7	12.0	5.6	41.0	1.9	0.13
	10.0	1.3	9.9	26.0	2.0	0.32

Hydrodynamic Radius and Diffusion Coefficient

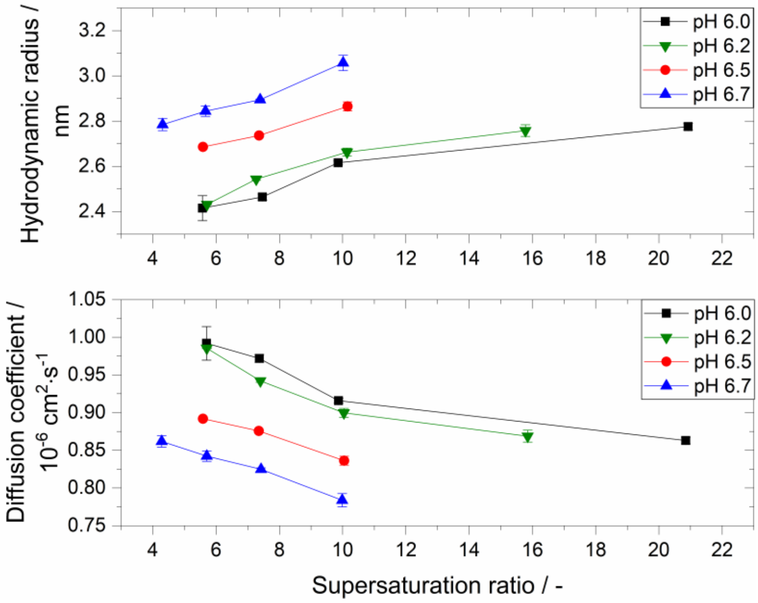

Figure 4. Hydrodynamic radius and diffusion coefficient of insulin at different solution pH as a function of supersaturation at 24.0 °C.

Determination of the Parameter A and B in the Classical Nucleation Equation

The values of A and B for homogeneous nucleation were obtained by plotting $\ln(J/n_0)$ vs $ln(S)^{-2}$ (see Figure 5). Within the plot of $\ln(J/n_0)$ vs $ln(S)^{-2}$ two regions can be identified: homogeneous nucleation (at a high supersaturation) and heterogeneous nucleation (at a low supersaturation). In the homogeneous nucleation regime $\ln(J/n_0)$ is decreasing linear with $ln(S)^{-2}$. By plotting a straight line through the data points within the homogeneous nucleation regime ($ln(S)^{-2}$ = 0.6 to 1.3) and neglecting the data points within the heterogeneous regime ($ln(S)^{-2}$ = 1.8) the values of the parameter A and B for homogeneous nucleation can be derived with the following correlation: $A = \exp\,(intercept)$ and $B = -slope$. Linear regression was used to obtain the parameter A and B for homogeneous insulin nucleation at different pH values (see Table 2).

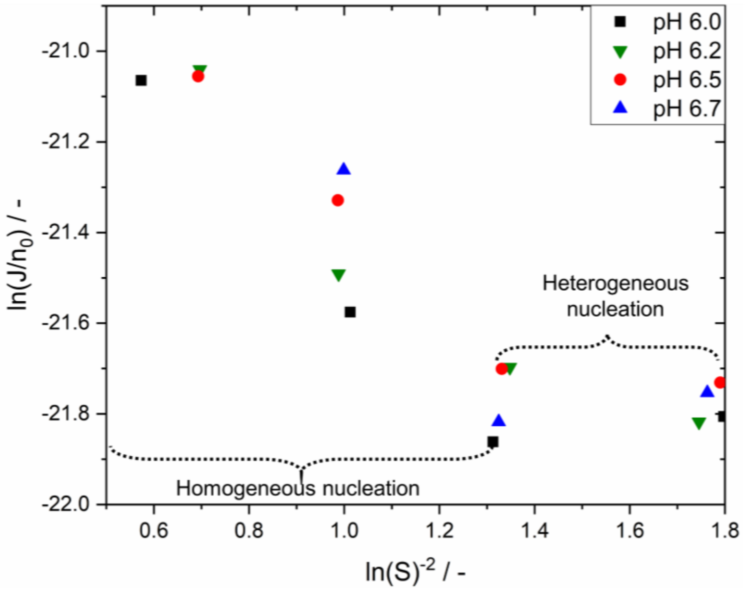

Figure 5. Dependencies of number density independent nucleation rate J/n_0 on thermodynamic supersaturation S at 24.0 °C.

Table 2. With linear regression obtained parameter A and B for classical nucleation equation for homogeneous nucleation of human insulin as function of solution pH at 24.0 °C.

pH	$Intercept\ /\ s^{-1}$	$A/\ 10^{-9}\ s^{-1}$	$Slope\ /\ -$	$B\ /\ -$
6.0	-20.47 ± 0.08	1.29 ± 007	-1.06 ± 0.08	1.06 ± 0.06
6.2	-20.41 ± 0.29	1.43 ± 0.40	-1.05 ± 0.23	1.05 ± 0.23
6.5	-20.34 ± 0.07	1.47 ± 0.06	-1.01 ± 0.07	1.01 ± 0.04
6.7	-21.82 ± 00	3.36 ± 0.00	-1.75 ± 0.00	1.75 ± 0.00

Validation of Setting Induction Time Equal to Latent Period to Derive the Parameter A and B in the Classical Nucleation Equation for Homogeneous Nucleation

As our objective was to obtain a relativity between the parameter A and B for homogeneous nucleation and the pH and not an absolute value, we would like to demonstrate that even at a shorter induction time the derivation of A and B via equation 5 still holds. To proof that latent period = induction time still holds, we calculated the parameter A and B for shorter induction times (e.g. 70 or 50%) as it can be seen in the tables below. Thereby "induction time = 0.7 * latent period" means, that if the measured latent period is 10.0 h the induction time until the first crystals were visible is 7.0 h. Although the values for A change slightly (max +35% at pH 6.5 for induction time = 0.5 * latent period), the trend is still the same: increase A with increasing pH. The values of B do not change. As we are only interested in the trend of A as function of pH (A increases with increasing pH) and not the absolute values, we believe that our evaluation of the induction time still holds.

Table 3. Induction time = latent period, this is equal to Table 2.

pH	$A/\ 10^{-9}\ s^{-1}$	$B\ /\ -$
6.0	1.29 ± 007	1.06 ± 0.06
6.2	1.43 ± 0.40	1.05 ± 0.23
6.5	1.47 ± 0.06	1.01 ± 0.04
6.7	3.36 ± 0.00	1.75 ± 0.00

Table 4. Induction time = 0.7 * latent period

pH	$A/\ 10^{-9}\ s^{-1}$	$B\ /\ -$
6.0	1.51 ± 0.13	1.06 ± 0.08
6.2	1.73 ± 0.41	1.05 ± 0.23
6.5	1.72 ± 0.12	1.01 ± 0.06
6.7	3.92 ± 0.00	1.75 ± 0.00

Table 5. Induction time = 0.5 * latent period

pH	$A/\ 10^{-9}\ s^{-1}$	$B\ /\ -$
6.0	1.75 ± 0.15	1.06 ± 0.08
6.2	2.00 ± 0.47	1.05 ± 0.23
6.5	1.99 ± 0.13	1.01 ± 0.06
6.7	4.54 ± 0.00	1.75 ± 0.00

Bibliography

[1] J. W. Mullin, *Crystallization*, 4th ed., Elsevier, **2001**.
